# Radiomics based on pretreatment MRI for predicting distant metastasis of nasopharyngeal carcinoma: A preliminary study

**DOI:** 10.3389/fonc.2022.975881

**Published:** 2022-08-09

**Authors:** Tingting Jiang, Yalan Tan, Shuaimin Nan, Fang Wang, Wujie Chen, Yuguo Wei, Tongxin Liu, Weifeng Qin, Fangxiao Lu, Feng Jiang, Haitao Jiang

**Affiliations:** ^1^ Department of Radiology, Cancer Hospital of the University of Chinese Academy of Sciences (Zhejiang Cancer Hospital), Hangzhou, China; ^2^ Institute of Basic Medicine and Cancer (IBMC), Chinese Academy of Sciences, Hangzhou, China; ^3^ Precision Health Institution, General Electric (GE) Healthcare, Hangzhou, China; ^4^ Department of Radiation Oncology, Cancer Hospital of the University of Chinese Academy of Sciences (Zhejiang Cancer Hospital), Hangzhou, China

**Keywords:** nasopharyngeal carcinoma, distant metastasis, radiomics, prediction model, magnetic resonance imaging

## Abstract

**Objective:**

To explore the feasibility of predicting distant metastasis (DM) of nasopharyngeal carcinoma (NPC) patients based on MRI radiomics model.

**Methods:**

A total of 146 patients with NPC pathologically confirmed, who did not exhibit DM before treatment, were retrospectively reviewed and followed up for at least one year to analyze the DM risk of the disease. The MRI images of these patients including T2WI and CE-T1WI sequences were extracted. The cases were randomly divided into training group (n=116) and validation group (n=30). The images were filtered before radiomics feature extraction. The least absolute shrinkage and selection operator (LASSO) regression was used to develop the dimension of texture parameters and the logistic regression was used to construct the prediction model. The ROC curve and calibration curve were used to evaluate the predictive performance of the model, and the area under curve (AUC), accuracy, sensitivity, and specificity were calculated.

**Results:**

72 patients had DM and 74 patients had no DM. The AUC, accuracy, sensitivity and specificity of the model were 0. 80 (95% CI: 0.72~0. 88), 75.0%, 76.8%, 73.3%. and0.70 (95% CI: 0.51~0.90), 66.7%, 72.7%, 63.2% in training group and validation group, respectively.

**Conclusion:**

The radiomics model based on logistic regression algorithm has application potential for evaluating the DM risk of patients with NPC.

## 1 Introduction

Nasopharyngeal carcinoma (NPC) is one of the most common head and neck cancers with a high incidence in South China, Southeast Asia, and North Africa (1). According to the latest data from the International Agency for Research on Cancer, the number of NPC patients from China in 2020 was 62444, of which 34,810 patients were died for the disease (2). More than 75% of patients were diagnosed with Locally advanced NPC (TNM stage III or IVA) at the first visit (3). Notably, due to highly sensitivity to radiotherapy, the prognosis of NPC has been greatly improved with advancements in radiotherapy and optimizations in chemotherapy regimens (4). At present, distant metastasis is still the major cause of treatment failure in NPC (5). As such, early diagnosis and accurate identification of DM is indispensable for timely implementation of reasonable treatment.

Currently, the anatomical tumor-node-metastasis (TNM) staging system is the main indicator for prognostic prediction, but this system has limitations in predicting DM and stratification for treatment decisions (6). Recent studies have shown that although patients within the same TNM stage received equivalent standard treatments, more than 20% of patients eventually developing DM showed poor efficacy and prognosis (7). The possible explanation is that TNM staging is mainly based on the anatomical information and cannot reflect the presence of heterogeneity in tumors. Hence, exploring an effective strategy to accurately identify patients at a high risk of DM is essential.

Radiomics, an emerging field of medical research, involves the transformation of traditional medical images into analyzable quantitative imaging features for model construction, and has shown great advantages in early diagnosis, efficacy evaluation, and prognosis prediction of tumors (8). This study aims to explore the feasibility of predicting distant metastasis risk (DM) of nasopharyngeal carcinoma (NPC) based on MRI radiomics model.

## 2 Materials and methods

### 2.1 Patients and datasets

In this retrospective study, the medical records and imaging data of NPC patients were obtained at Zhejiang Cancer Hospital from January 2010 to December 2016. The clinical features including gender, age, T stage, and histological types were collected. Inclusion criteria were: (1) two sequences (axial T2-weighted [T2WI] and T1-weighted contrast [CE-T1WI]) of head and neck MRI were all collected; (2) all patients were diagnosed with NPC by pathology and did not exhibit DM before treatment; (3) all patients were followed up for more than one year, with a maximum of 6 years; (4) no prior malignancy. Exclusion criteria were: (1) received antitumor therapy before MRI examination; (2) artifacts in MRI images; (3) the tumor is too small (there is volume effect when sketching the target ROI). Finally, a total of 146 NPC patients were included, all patients were randomly divided into training group (n = 116) and validation group (n = 30) as a ratio of 4:1 by computer. This study was approved by the Ethics Committees of Zhejiang Cancer Hospital.

### 2.2 Image acquisition

MRI was performed with Siemens 3.0T MR scan equipment and a 16-channel head and neck joint coil. Scanning sequence and parameters: (1) axial T1-weighted imaging (T1WI): TR: 498ms, TE: 8ms, slice thickness: 5mm, FOV: 260×260mm, matrix size: 288×229. (2) axial T2-weighted imaging (T2WI): TR: 3020ms, TE: 100ms, slice thickness: 4mm, FOV: 260×260mm, matrix size: 372×363. The contrast medium was Gd-DTPA, dose 15mL (0.1mmol/kg), injection rate 2.0mL/s. Contrast enhanced T1WI (CE-T1WI scan was performed after 1min after intravenous injection of the elbow.

### 2.3 Research methods of radiomics

Tumor segmentation: T2WI and CE-T1WI images were introduced into ITK-SNAP software (version 3.8.0, http://www.itksnap.org/) for tumor segmentation. Two doctors (with more than 8 years of experience in neck diagnosis, respectively) manually sketched the target ROI layer by layer, and selected axial images of each sequence to avoid enlarged lymph nodes in parapharyngeal space as far as possible.

**Figure 1:**
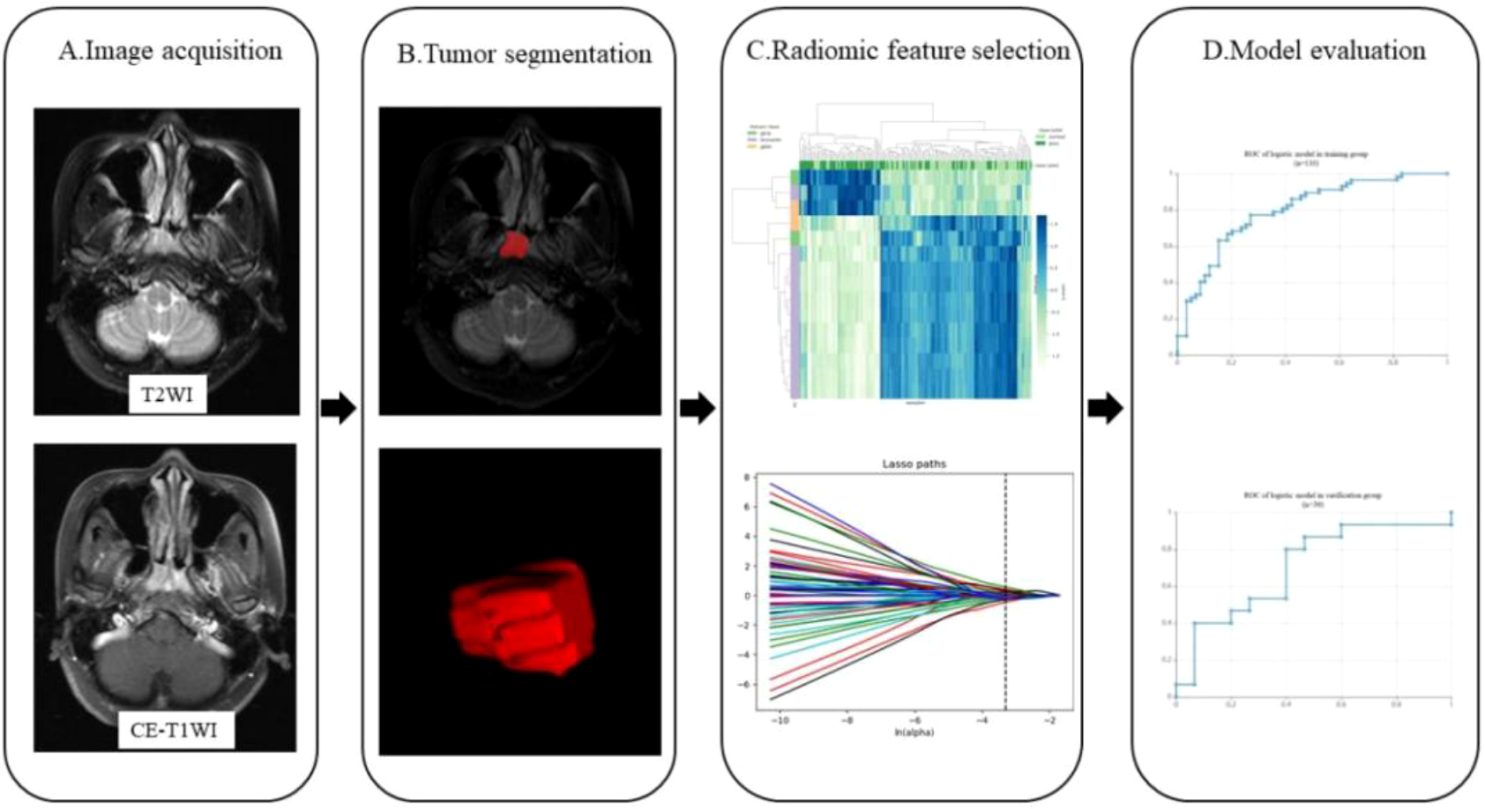
Workflow showing the establishment of a radiomics model based on MRI for predicting DM of NPC. The steps include **(A)** MR image acquisition, **(B)** tumor segmentation, **(C)** radiomics features selection, and **(D)** model evaluation.

Radiomics feature extraction and radiomics model building: all the segmented ROI data were imported into the Darwin research platform for feature extraction. The definition and calculation formula of features are in line with the PyRadiomics standard (9). In order to avoid reducing the speed of calculation, the extracted features were standardized by the minimum and maximum scaling algorithm. The optimal feature selection was used for removing the low performance features, the K was set to 15% and the f calssif function was selected which refers to the first 15% features sorted by F value were selected by the analysis variance of F test statistics. The minimum absolute contraction and selection operator (LASSO) regression was used to further reduce the dimension of the feature parameters. Finally, 15 combinatorial features were obtained. Logistic regression algorithm was used to establish a model includes above selected feature parameters. The diagnostic efficacy evaluation of the training group and validation group model were obtained from the receiver operating characteristic (ROC) and area under curve (AUC), accuracy, sensitivity, and specificity. The workflow of the radiomics procession is presented in **Figure 1**.

### 2.4 Statistical analysis

The general characteristics of patients were statistically analyzed by SPSS 26.0. The classified data was compared by chi-square test or Fisher’s exact test. The independent sample t-test was applied in the analysis of the quantitative data which according to normal distribution were expressed as mean ± standard deviation (). The Mann-Whitney U test was used for the comparison of quantitative data which did accord to normal distribution. *P*< 0.05 indicates that the difference is statistically significant.

## 3 Results

### 3.1 Clinical characteristics of the patients

A total of 146 patients with NPC were collected and followed up for at least one year, of which 72 patients had DM, and other 74 patients had no DM. Histological subtypes were divided into three types: type I differentiated keratinizing carcinoma (n=15), type II differentiated nonkeratinizing carcinoma (n=69), and type III undifferentiated nonkeratinizing carcinoma (n=62). There was a significant difference in T-stage between the two groups (*P*< 0.001). The T stage in DM group was higher as a whole. There was no significant difference in sex, age and histological types between the two groups (*P*> 0.05), as shown in **Table 1**.

**Table 1:** Comparison of general characteristics of patients with NPC.

	DM group (n=72)	Non-DM group (n=74)	Statistical value	*P*
Gender (male/female)	58/16	54/20	0.744	0.388^b^
age	47.9 ± 12.5	48.6 ± 12.9	0.362	0.718^a^
Tumor stage			5.943	<0.001^c^
T1	3	15		
T2	13	35		
T3	25	18		
T4	31	6		
Histological type			0.658	0.510^c^
I type	6	9		
II type	34	35		
III type	32	30		

a: t value; b: x^2^ value; c: Z value.

### 3.2 Radiomics feature selection results

**Figure 2:**
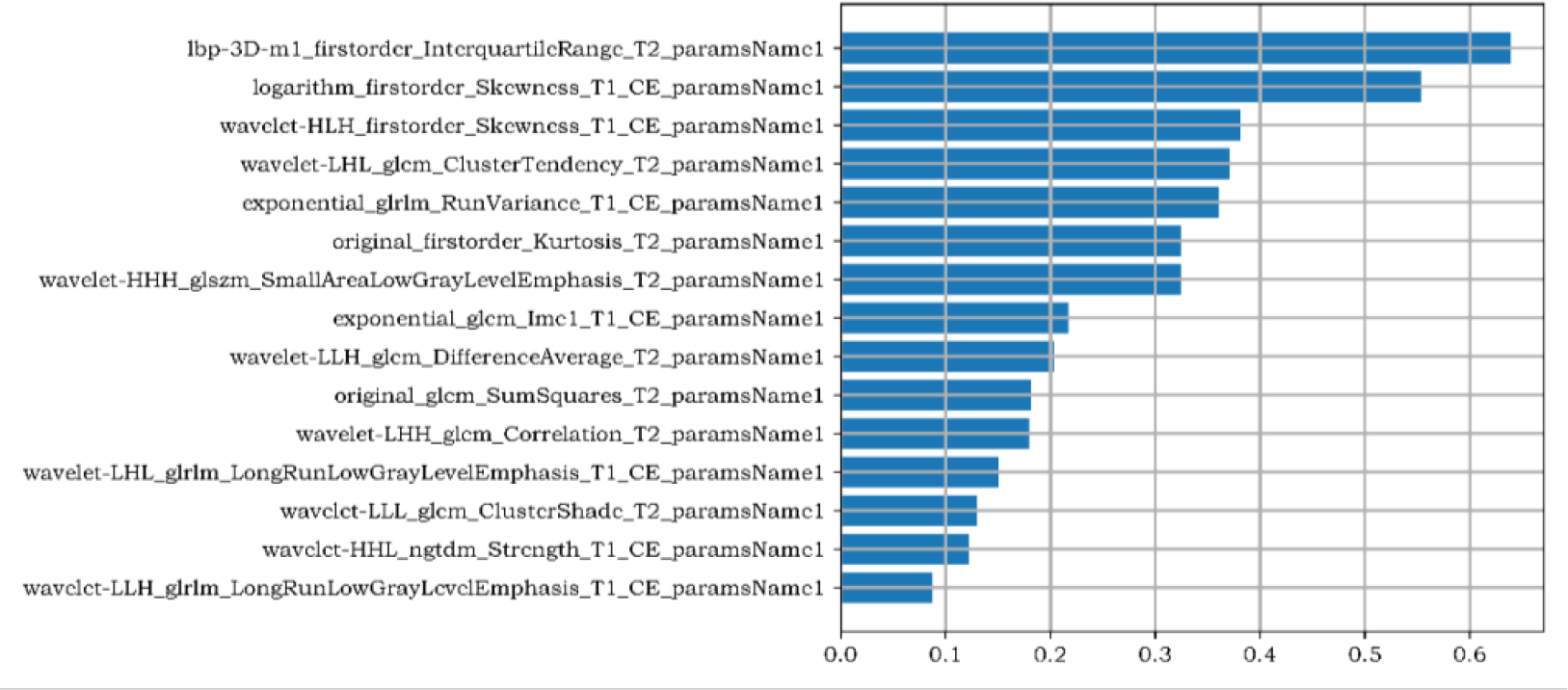
The Gini Coefficient importance analysis of radiomics features. The three radiomics features with the highest contribution are the inter quartile range of the first-order feature, the skewness of the first-order feature after logarithm, and the skewness of the first-order feature filtered by high-low- high wavelet filters in XYZ direction,.

1781 radiomics features were extracted from magnetic resonance images: (1) first-order features, (2) shape features, (3) texture features: gray level co-occurrence matrix (GLCM), gray level run length matrix (GLRLM), gray level size zone matrix (GLSZM), gray level dependence matrix (GLDM), neighbouring gray tone difference matrix (NGTDM). There were 15 most valuable imaging features after dimensionality reduction with LASSO, including first-order features (n=4) and texture features (n=11). The first-order features including: inter quartile range, skewness (n=2), kurtosis. GLCM features include: cluster tendency, difference average (DA), sum squares (SS), correlation, cluster shade (CS), informational measure of correlation (Imc1). GLRLM features include: run variance (RV), long run low gray level emphasis (LRLGLE) (n=2). GLSZM features including: small area low gray level emphasis (SALGLE); NGTDM features including: strength. The Workflow of radiomics model was shown in **Figure 2**.

### 3.3 Prediction model results

**Figure 3:**
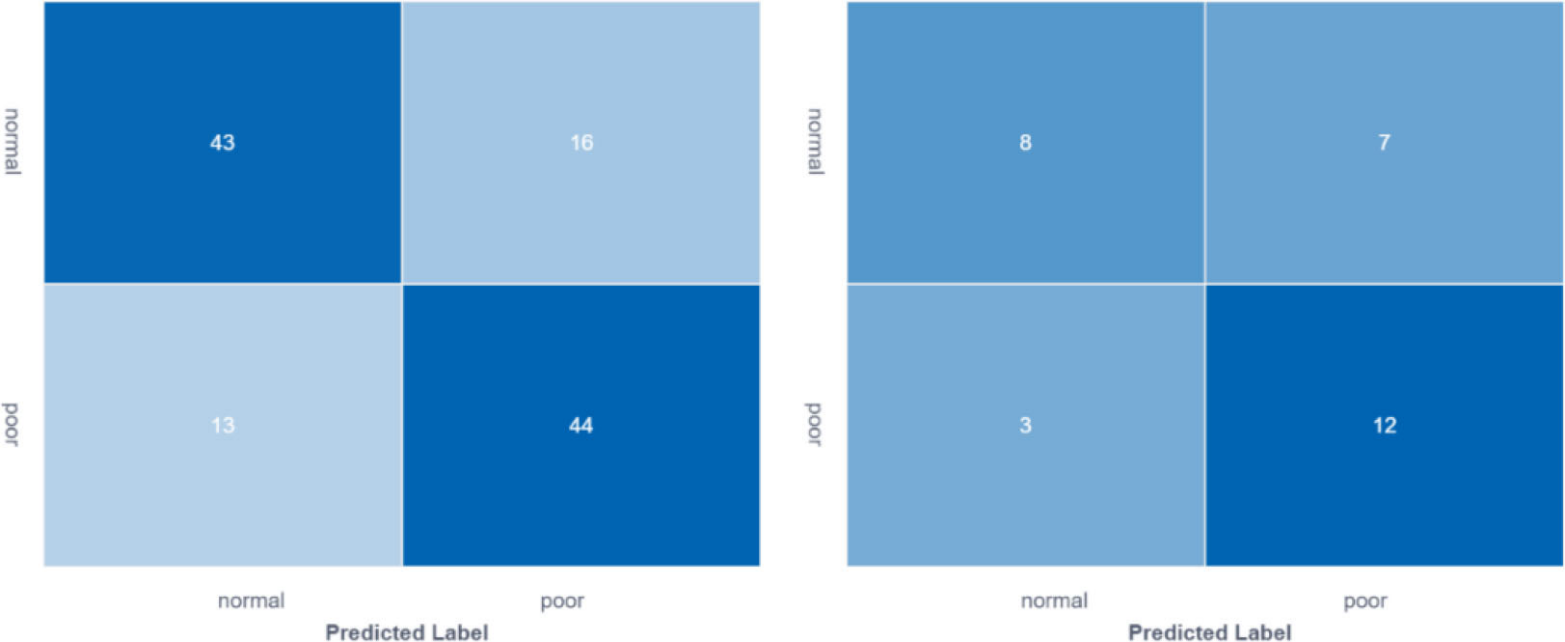
Comparison of feature model cross-validation performance between training group and validation group.

**Figure 4:**
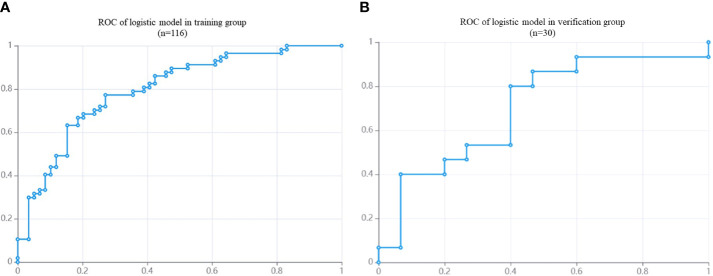
The ROC of DM in NPC patients based on MRI radiomics model. **(A)**: the AUC of training group (n = 116) is 0.80. **(B)**: the AUC of validation group (n = 30) is 0.70.

In the training group, AUC was 0.80 (95% CI: 0.72- 0.88), the sensitivity was 76.8%, the specificity was 73.3% and the accuracy was 75.0%. In the validation group, the AUC value of the model was 0.70 (95% CI: 0.51- 0. 90), the sensitivity was 72.7%, the specificity was 63.2% and the accuracy was 66.7%. The corresponding characteristic coefficients and the comparison of characteristic parameters between the two groups were shown in **Table 2**, **Figures 3**, **4**.

**Table 2:** Predictive effectiveness of radiomics model in the training group and validation group.

	training group (n=116)	validation group(n=30)
AUC (95% CI)	0. 80(0.72~0. 88)	0.70(0.51~0. 90)
sensitivity	76.8%	72.7%
specificity	73.3%	63.2%
accuracy	75.0%	66.7%

## 4 Discussion

MRI is one of the most commonly used in the early diagnosis and evaluation of NPC, and its sensitivity and resolution of lesions are better than CT images. Although radiotherapy and simultaneous radiotherapy and chemotherapy significantly reduced the local recurrence rate of NPC, the treatment response of patients with DM was poor, and the 5-year survival rate was less than 5%, which was the main cause of treatment failure (10). Therefore, it is necessary to evaluate the risk of metastasis in patients with NPC before treatment in order to adopt more aggressive therapy strategies for high-risk patients. MRI only simply reflected the anatomical structure of tumor invasion, but ignored the heterogeneity of tumor. It was difficult to monitor and evaluate the risk of tumor patients with DM during treatment, which has many limitations. By extracting and analyzing medical images and obtaining quantitative feature data that cannot be recognized by the naked eye, the imaging model can more comprehensively and carefully show the microscopic characteristics and heterogeneity of the tumor. Up to now, few studies have used imaging analysis to predict the occurrence of DM in NPC (11, 12). Most of them used imaging models to predict tumor stage, recurrence, curative effect evaluation and prognosis evaluation (13–18). Zhang (12) reported that combined MRI imaging features with clinical features to evaluate DM risk in patients with NPC before the first treatment, and found that the combined model had good diagnostic efficacy in both training group and validation group. Peng (11) found that the combination of sequence floating forward selection (SFFS) and support vector machine (SVM) classifier can further improve the accuracy of imaging prediction model by analyzing the characteristics of preprocessed PET/CT images for prediction the recurrence and DM in patients with locally advanced NPC.

In this study, we extracted the imaging features of T2WI and CE-T1WI sequences in MR images and constructed a radiomics model based on logistic regression to predict the risk of DM in NPC patients before initiating treatment. The model has higher AUC value, sensitivity, specificity, and accuracy in the training group and validation group, suggesting that it has a good diagnostic performance. Similarly, previous studies have found that the ability of image group feature prediction based on T2WI and CE-T1WI image extraction was better than that of independently T2WI sequence or independently CE-T1WI sequence image extraction (19). T2WI images mainly provide anatomical information, and CE-T1WI images mainly evaluate the blood supply of tumor. The combination of two sequence images is a key factor in judging the prognosis of NPC.

In this study, 1781 radiomics features were extracted. After dimensionality reduction with LASSO, 15 radiomics features were remained, including first-order features (n=4) and second-order GLCM (n=6), GLRLM (n=3), GLSZM (n=1) and NGTDM (n=1). The first-order feature mainly describes the distribution of voxel gray values in ROI. The second-order histogram feature or texture feature is a feature describing the spatial distribution intensity level of voxels. GLCM describes the joint distribution of two gray pixels with a certain spatial position relationship, in which the correlation is the feature extracted from the T2WI image, which reflects the consistency of the image texture. The greater the value difference, the higher the heterogeneity in the tumor. Cluster Tendency is a measure of groupings of voxels with similar gray-level values. DA refers to the relationship between similar intensity values and different intensity values. SS quantifies the distribution of neighbouring intensity level pairs about the mean intensity level. Correlation represents the linear dependency of gray level values to their respective voxels. CS is a measure of the skewness and uniformity of the GLCM: a higher CS implies greater asymmetry about the mean. GLRLM is a quantitative index of the smoothness of image texture, in which the larger the LRLGLE value, the higher the gray value and the smoother of the texture in image. RV is a measure of the variance in runs for the run lengths. Similar to GLRLM, GLSZM, mainly describes the quantitative index of image texture uniformity: SALGLE refers to the proportion in the image of the joint distribution of smaller size zones with lower gray-level values, and this feature is positively correlated with tumor heterogeneity. NGTDM refers to the sum of the difference between a gray value and the average gray value of its neighbours: texture intensity is related to contrast and coarseness, with small values for coarseness textures and high values for busyness or fine textures. Strength is a measure of the primitives in an image. Its value is high when the primitives are easily defined and visible. These features are objective and quantitative information that cannot be observed by the human eye, and usually reflect the pathophysiological information inside the tumor. The results of this study showed that the differences in the characteristic parameter values between the DM group and the non-DM group were in line with the above rules, and the overall texture distribution of the tumors in the DM group was uneven, that is, the inherent heterogeneity of tumor may provide additional information about pathophysiological features.

There were several limitations in the present study: (1) this study is a retrospective analysis of single-center with a small sample size, which needs to be verified by multicenter, prospective studies with a large sample size, (2) the thick slice thickness (5mm) of the images used in this study may have effects on ROI segmentation, (3) different types of MRI equipment are used for inspection, the scanning parameters are not unified.

## 5 Conclusions

Radiomics analysis can objectively quantify the morphological and internal heterogeneity changes of NPC, and the radiomics model can effectively evaluate the risk of DM in patients with NPC, which may assist physicians in screening patients with DM and accordingly formulating individualized treatment plans for patients.

## Data availability statement

The raw data supporting the conclusions of this article will be made available by the authors, without undue reservation.

## Ethics statement

The studies involving human participants were reviewed and approved by Ethics Committees of Zhejiang Cancer Hospital. Written informed consent for participation was not required for this study in accordance with the national legislation and the institutional requirements.

## Author contributions

TJ had full access to all of the data in the study and took responsibility for the integrity of the data and the accuracy of the data analysis. Collection and assembly of data: TJ, YT, SN, WC, FW, TL, FL. Resources and data curation: TL, WQ, FJ. Data analysis and interpretation: TJ, YW, HJ, FW. Writing-original draft preparation: TJ. Statistical analysis: TJ, YW, HJ. Study concept, supervision, funding acquisition, project administration, writing-review: HJ and FJ. All authors contributed to the article and approved the submitted version.

## Funding

This study was supported by grants from Medical and Health Research Project of Zhejiang Province (Grant Number: 2020KY486), and Natural Science Foundation of Zhejiang Province (2022C03072).

## Conflict of interest

Author YW was employed by General Electric (GE) Healthcare.

The remaining authors declare that the research was conducted in the absence of any commercial or financial relationships that could be construed as a potential conflict of interest.

## Publisher’s note

All claims expressed in this article are solely those of the authors and do not necessarily represent those of their affiliated organizations, or those of the publisher, the editors and the reviewers. Any product that may be evaluated in this article, or claim that may be made by its manufacturer, is not guaranteed or endorsed by the publisher.
